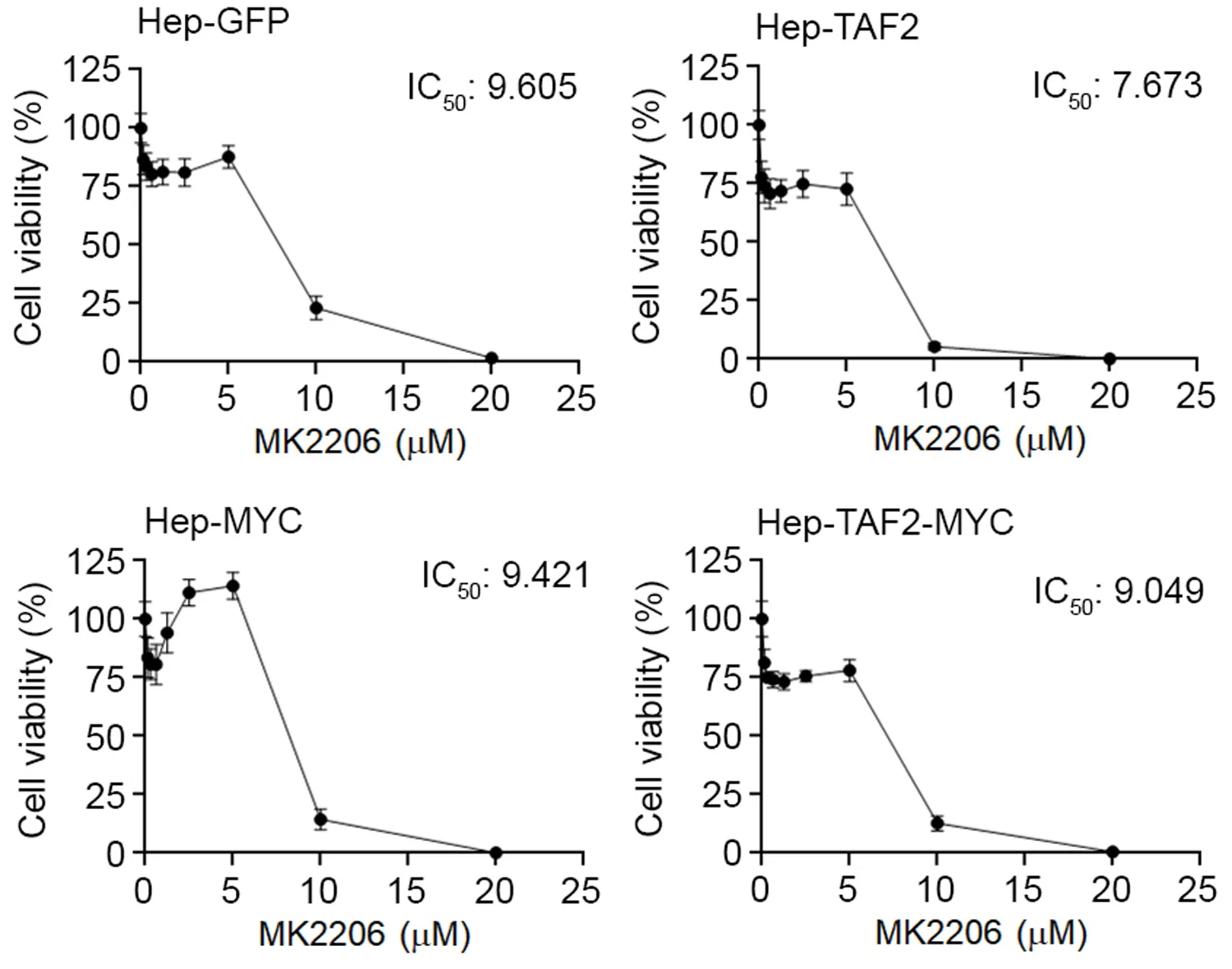# Molecular mechanism of augmentation of oncogenic activity of MYC by TAF2 in HCC

**DOI:** 10.1016/j.jbc.2026.113581

**Published:** 2026-09-18

**Authors:** Suchismita Raha, Qiong Zhang, Devon Farrar, Charles Tseng, Rachel G. Mendoza, Rabha M. Younis, Kayla A. Rodriguez, Samyukta Iyer, Mark A. Subler, Jolene J. Windle, Zhao Lai, Mikhail Dozmorov, Jamy C. Peng, Devanand Sarkar

**Affiliations:** 1 Department of Cellular, Molecular and Genetic Medicine, Virginia Commonwealth University, Richmond, Virginia, USA; 2 Center for Applied Bioinformatics, St Jude Children’s Research Hospital, Memphis, Tennessee, USA; 3 Department of Developmental Neurobiology, St Jude Children’s Research Hospital, Memphis, Tennessee, USA; 4 Greehey Children’s Cancer Research Institute, University of Texas Health Science Center San Antonio, San Antonio, Texas, USA; 5 Departments of Biostatistics and Pathology, Virginia Commonwealth University, Richmond, Virginia, USA; 6 Massey Comprehensive Cancer Center, Virginia Commonwealth University, Richmond, Virginia, USA

**Keywords:** HCC, TAF2, MYC, ras, gene regulation, MEK inhibitor

## Abstract

The oncogenes MYC and TAF2 (TATA-box binding protein associated factor 2) are frequently overexpressed and co-amplified in many cancers, including hepatocellular carcinoma (HCC). We recently demonstrated that overexpression of TAF2 in mouse liver markedly augmented MYC overexpression-induced HCC. MYC is a transcription factor. TAF2 is a component of transcription factor IID (TFIID) and functions as a transcription co-factor. We hypothesized that TAF2 modulates gene regulation by MYC, contributing to augmentation of MYC’s oncogenic activity. To test this hypothesis, we generated stable HepG2 cell lines overexpressing TAF2, MYC, or both. Combined overexpression of TAF2 and MYC significantly augmented *in vitro* proliferation, migration and invasion, compared to the overexpression of each gene alone. We performed RNA-sequencing and CUT&RUN (cleavage under targets & release using nuclease) to identify MYC-regulated genes that are induced only by TAF2 overexpression. RASGEF1A (RasGEF domain family member 1A), a guanine exchange factor activating Ras, was significantly induced by TAF2 and MYC overexpression. Consequently, the MEK/ERK pathway was robustly activated in cells co-overexpressing TAF2 and MYC, rendering them uniquely sensitive to the MEK inhibitor Trametinib. These findings suggest that HCC patients with TAF2 and MYC overexpression might benefit from targeted treatment with MEK inhibitors.

Hepatocellular carcinoma (HCC) is a highly fatal disease, and its incidence and mortality rates have been alarmingly increasing (1, 2). Currently, the most effective Food and Drug Administration (FDA)-approved treatment for advanced non-resectable HCC is a combination of anti-programmed cell death ligand-1 (anti-PD-L1; atezolizumab) and anti-vascular endothelial growth factor-A (anti-VEGFA; bevacizumab) antibodies, which provide an overall response rate of 27% (3, 4). This bleak scenario mandates that novel players regulating HCC be identified that could be targeted to develop effective combinatorial therapies. Amplification of chromosome 8q is a common event in cancers, including HCC (5, 6). An important oncogene in this amplicon is MYC (encoding c-Myc) at 8q24.21. MYC is a transcription factor that heterodimerizes with MAX to bind to E-box sequence (CACGTG) in the promoters of target genes, which regulate proliferation, differentiation, cell cycle, metabolism, apoptosis, angiogenesis, and metastasis. In molecular subclasses of HCC, the S2 subclass is characterized by a MYC signature and poor survival (7). MYC overexpression induces HCC while antisense inhibition reverses HCC in mice (8, 9, 10, 11, 12).

TATA-box binding protein associated factor 2 (TAF2), residing in 8q24.12, is co-amplified with MYC in ∼18% of human patients with HCC in The Cancer Genome Atlas (TCGA) database (13). TAF2 is a component of the general transcription factor TFIID complex (14, 15), thereby participating in regulation of basal transcription. In our recent study, we used Sleeping Beauty (SB) transposase-mediated somatic integration in combination with the hydrodynamic tail vein injection (HTVI) technique in FVB/N mice to overexpress human TAF2 and MYC in normal mouse liver (13). Five weeks after injection, none of the control mice developed tumors, and only one mouse in the TAF2 group showed a single <1 mm tumor (13). Overexpression of MYC alone resulted in tumor development at varying degrees in all mice (13). The combination of TAF2 and MYC robustly and significantly increased HCC development compared to MYC overexpression alone (13). However, the molecular mechanism by which TAF2 promotes MYC-induced hepatocarcinogenesis remained to be determined.

Given that MYC is a transcription factor and TAF2 is a transcription co-factor, we reasoned that overexpression of TAF2 modulates MYC-mediated gene regulation, thereby accentuating its oncogenic property. In this study, we employed an *in vitro* system to show that TAF2 facilitates MYC-mediated expression of specific oncogenes, which results in activation of MEK/ERK pathway. This activation confers a unique sensitivity to MEK inhibitors in HCC cells overexpressing both TAF2 and MYC.

## Results

We previously demonstrated that in mouse liver, overexpression of TAF2 alone did not induce tumorigenesis, but it markedly augmented overexpression of MYC-induced HCC (13). MYC is a transcription factor, and TAF2 is a transcription co-factor. We, therefore, hypothesized that overexpression of TAF2 might modulate MYC-regulated gene expression and thereby increase the oncogenic function of MYC. To test this hypothesis, we generated an *in vitro* system in which we established stable clones in HepG2 cells overexpressing GFP (control), TAF2, MYC and both TAF2 and MYC (Hep-GFP, Hep-TAF2, Hep-MYC and Hep-TAF2-MYC, respectively). MYC overexpression induces apoptosis (16). We endeavored to establish similar overexpressing clones in additional human HCC cells, but apart from HepG2 cells, MYC-overexpressing clones in other HCC cells eventually died out. TAF2 and MYC constructs harbor a V5- and an HA-tag in their C-terminus, respectively. The authenticity of the clones was confirmed by Western blot in which TAF2-V5 was detected in Hep-TAF2 and Hep-TAF2-MYC clones, and MYC-HA was detected in Hep-MYC and Hep-TAF2-MYC clones (Fig. 1*A*). Compared to Hep-GFP clone, proliferation (determined by MTT and colony formation assay), invasion (determined by Matrigel invasion assay), and migration (determined by wound healing assay) were significantly increased in Hep-TAF2 and Hep-MYC clones, which were further augmented in Hep-TAF2-MYC clone (Fig. 1, *B*–*G*). These findings indicate that although both TAF2 and MYC could promote cancer hallmarks, together they exerted a potent additive effect.

**Figure 1:**
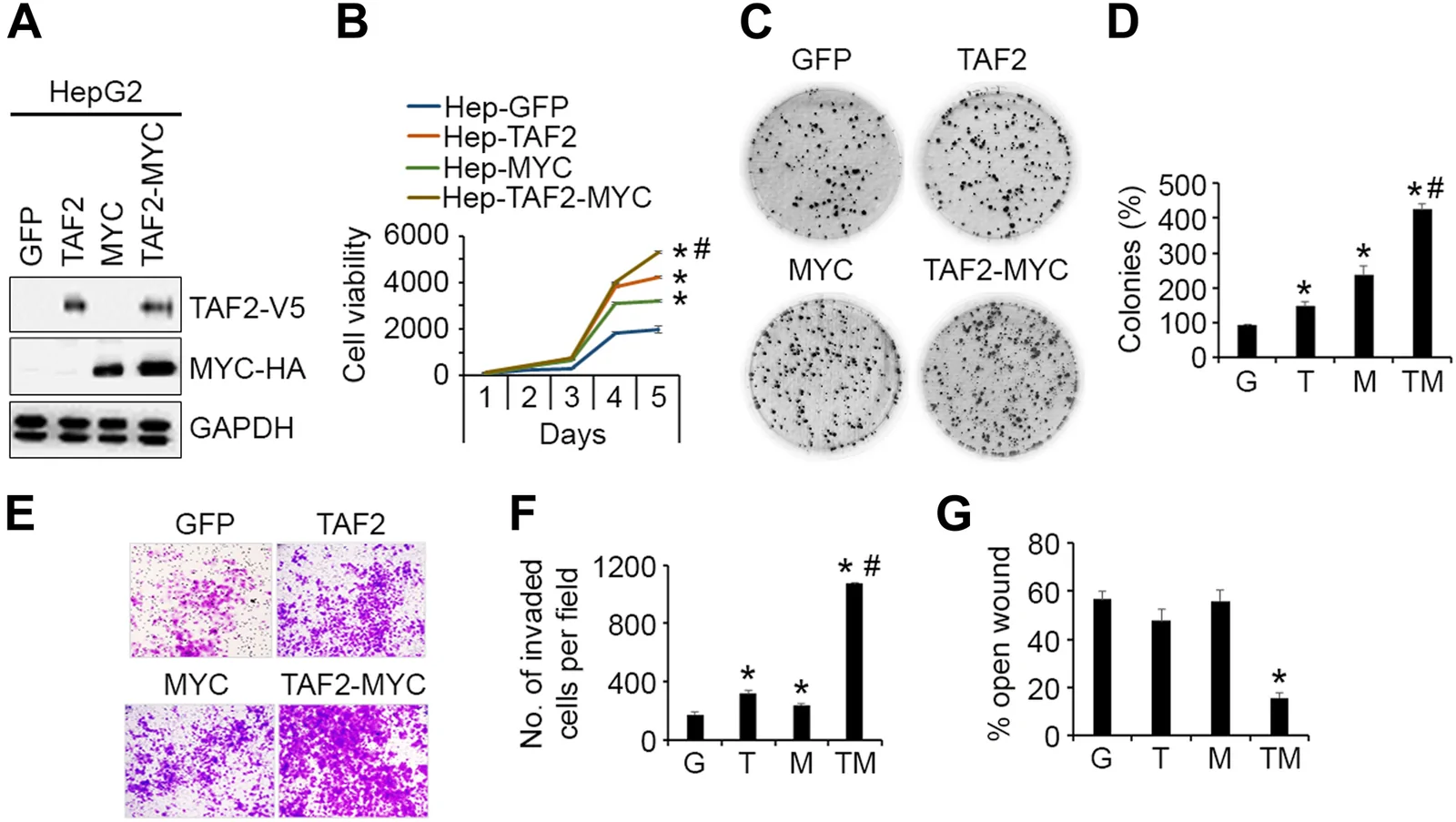
**Overexpression of TAF2 and MYC in HepG2 cells significantly augments cancer hallmarks.***A*, representative Western blot using anti-V5 and anti-HA antibodies in the indicated clones. GAPDH was used as loading control. *B*, cell viability of the indicated clones determined by MTT assay. Viable cells at day 1 was considered as 100. *C*, representative images of colony formation assay performed in the indicated clones. *D*, quantification of the data of the colony formation assay. *E*, representative photomicrographs of Matrigel invasion assay performed in the indicated clones. *F*. Quantification of the data of Matrigel invasion assay. For, *B*, *D* and *F*, data represent mean ± SEM. ∗: *p* < 0.01 *versus* Hep-GFP; #: *p* < 0.01 *versus* Hep-TAF2 and Hep-MYC. *G*, Wound healing assay was performed in the indicated clones. Quantification of the percent of open wound after 48 h. Data represent mean ± SEM. ∗: *p* < 0.01 *versus* the other three groups. G, Hep-GFP; M, Hep-MYC; T, Hep-TAF2; TM, Hep-TAF2-MYC.

Both TAF2 and MYC regulate transcription. We checked colocalization of TAF2 and MYC by performing dual immunofluorescence (IF) analysis using anti-V5 (green for TAF2) and anti-HA (red for MYC) antibodies (ab) in the Hep-TAF2-MYC clone (Fig. 2*A*). TAF2 and MYC were located in the nucleus and the yellow color in the merged image indicates that they co-localize (Fig. 2*A*). We performed co-immunoprecipitation (co-IP) experiment in the Hep-TAF2-MYC clone, where we performed IP with anti-HA ab and immunoblotted (IB) with anti-V5 ab and *vice versa*. Anti-HA ab, but not control IgG, efficiently pulled down TAF2-V5, and anti-V5 ab efficiently pulled down MYC-HA, confirming that MYC and TAF2 interact with each other (Fig. 2*B*). To check the interaction between TAF2 and MYC in an additional HCC cell line, we performed co-IP in the human HCC cell HuH-7 where IP was done with anti-MYC ab, and IB was done with anti-TAF2 ab and *vice versa*. The levels of TAF2 and MYC in parental HepG2 and HuH-7 cells are shown in Fig. S1*A*. Anti-MYC ab efficiently pulled down TAF2, and anti-TAF2 ab efficiently pulled down MYC, confirming interaction of endogenous TAF2 and MYC (Fig. 2*C*). In HuH-7 cells, endogenous interaction of MYC with other TFIID components, such as TBP and TAF8, was also observed by co-IP assays (Fig. S1*B*).

**Figure 2:**
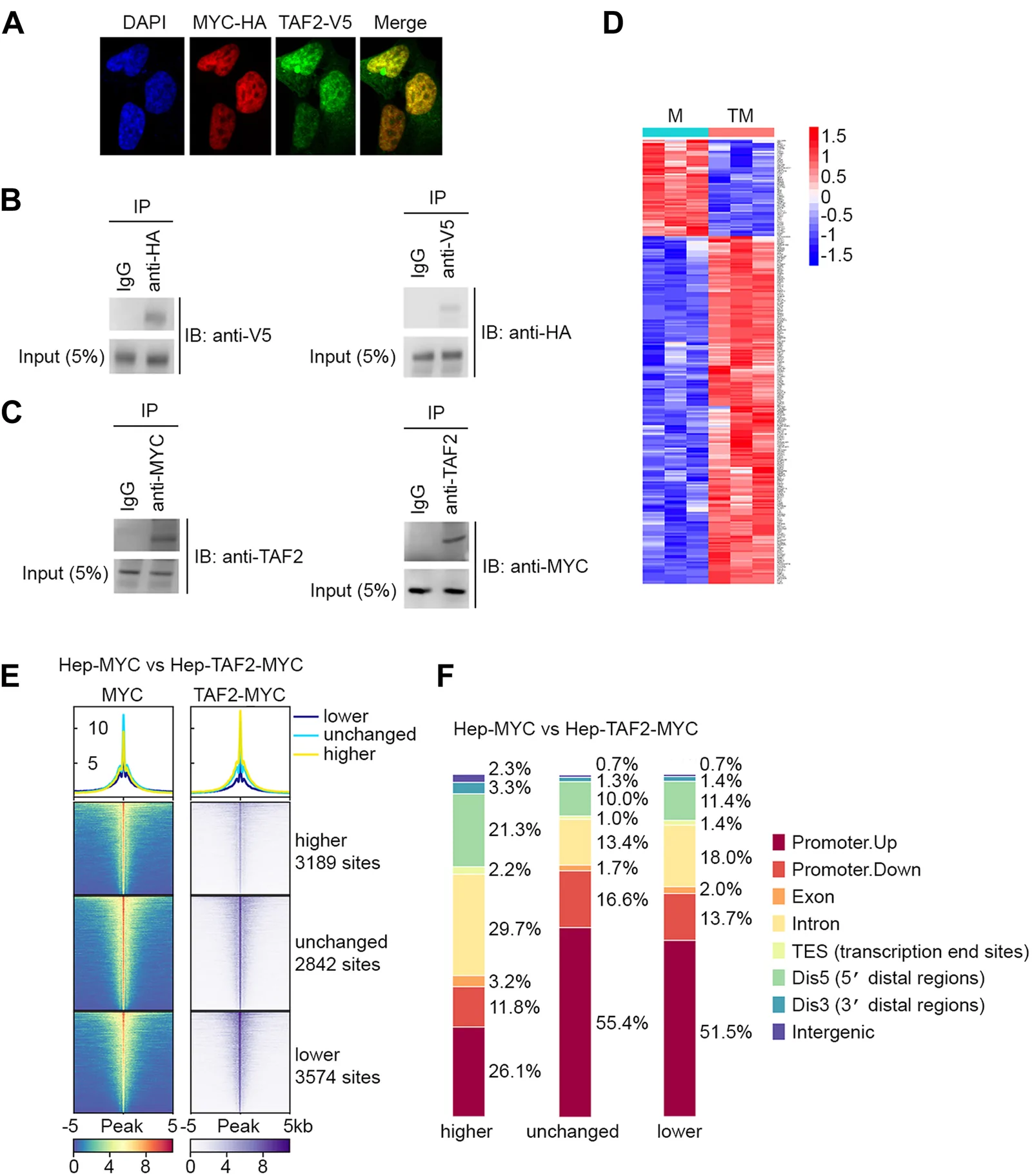
**TAF2 overexpression alters MYC-mediated gene expression.***A*, representative photomicrographs of double immunofluorescence analysis using anti-V5 (*green*) and anti-HA antibodies (*red*) in Hep-TAF2-MYC clone. The merged image shows co-localization of TAF2 and MYC (*yellow*) in the nucleus. *B*. Lysates from Hep-TAF2-MYC clone were immunoprecipitated (IP) with anti-HA antibody and immunoblotted (IB) with anti-V5 antibody, and *vice versa*. *C*, Lysates from HuH-7 cells were immunoprecipitated (IP) with anti-MYC antibody and immunoblotted (IB) with anti-TAF2 antibody and *vice versa*. *D*, heatmap showing differentially expressed genes (DEGs) in Hep-MYC and Hep-TAF2-MYC clones by RNA-seq. *E*, heatmaps align MYC-HA CUT&RUN peaks for Hep-MYC and Hep-TAF2-MYC clones. Average profiles represent genomic sites with lower, unchanged, or higher MYC-HA signals in Hep-MYC compared to Hep-TAF2-MYC. Statistical significance was determined using an FDR-adjusted *p* < 0.05. *F*. Proportion of MYC binding sites to different genomic features in Hep-MYC *versus* Hep-TAF2-MYC clones as revealed by CUT&RUN analysis.

We compared global gene expression between Hep-TAF2-MYC and Hep-MYC clones by performing RNA-sequencing (RNA-seq). In this analysis, using a log2 fold-change (log2FC) of 0.5 and false discovery rate (FDR) of 0.01 cut-off, we identified 278 genes which were differentially expressed in Hep-TAF2-MYC clone compared to Hep-MYC clone (Fig. 2*D* and Table S1). Out of these 278 genes, 218 were upregulated and 60 were downregulated. Simultaneously, we performed Cleavage Under Targets & Release Using Nuclease (CUT&RUN) in these clones using HA antibody to identify binding of MYC in Hep-MYC cells compared to MYC binding when TAF2 is overexpressed (Hep-TAF2-MYC). For CUT&RUN peak calling, Hep-GFP clone was used as control for Hep-MYC and Hep-TAF2-MYC clones. Two replicate data for each group were merged for analysis, using FDR-adjusted *p* < 0.05 cut-off to identify differential binding of MYC with endogenous *versus* overexpressed TAF2 (Fig. 2*E*). Annotation of MYC binding sites to genetic features showed that MYC is highly enriched at promoters (Fig. 2*F*). TAF2 overexpression led to lower MYC binding at introns and distal regions and higher MYC binding at promoters (Fig. 2*F*). As total protein level of MYC is not changed by TAF2 overexpression (Fig. 1*A*), CUT&RUN data suggest that TAF2 changes MYC binding from specific introns and distal regions to specific promoters.

In the CUT&RUN analysis, using a log2FC of 1 and an adjusted *p*-value of 0.05, differential MYC binding was identified at 1437 sites in 1081 genes in the Hep-TAF2-MYC clone compared to the Hep-MYC clone (Table S2). Out of these 1437 sites, 586 showed positive MYC-binding and 851 showed negative MYC-binding. For a number of genes, differential MYC binding was observed in different regions of the same gene, including promoter, intron and distal regions (Table S2). We performed Ingenuity Pathway Analysis (IPA) using all 1437 differential MYC binding sites between Hep-TAF2-MYC and Hep-MYC clones. Figure 3*A* shows a graphical summary of genes, pathways and phenotypes activated or inhibited in the Hep-TAF2-MYC clone compared to the Hep-MYC clone. There was marked activation of cell transformation, proliferation, invasion and metastasis, along with activation of oncogenes, such as CREB binding lysine acetyltransferase (CREBBP/CBP), erb-b2 receptor tyrosine kinase 2 (ERBB2/HER2), signal transducer and activator of transcription 3 (STAT3), TEA domain transcription factor 4 (TEAD4) and transforming growth factor beta 1 (TGFB1), in Hep-TAF2-MYC clone compared to Hep-MYC clone (Fig. 3*A*). Inflammatory signaling, driven by tumor necrosis factor alpha (TNF) and TNF receptor 1 (TNFR1) as well as REL proto-oncogene, NF-κB subunit (REL), was significantly activated in the Hep-TAF2-MYC clone *versus* the Hep-MYC clone (Fig. 3*A*). These findings correlate with the phenotypes observed in Figure 1.

**Figure 3:**
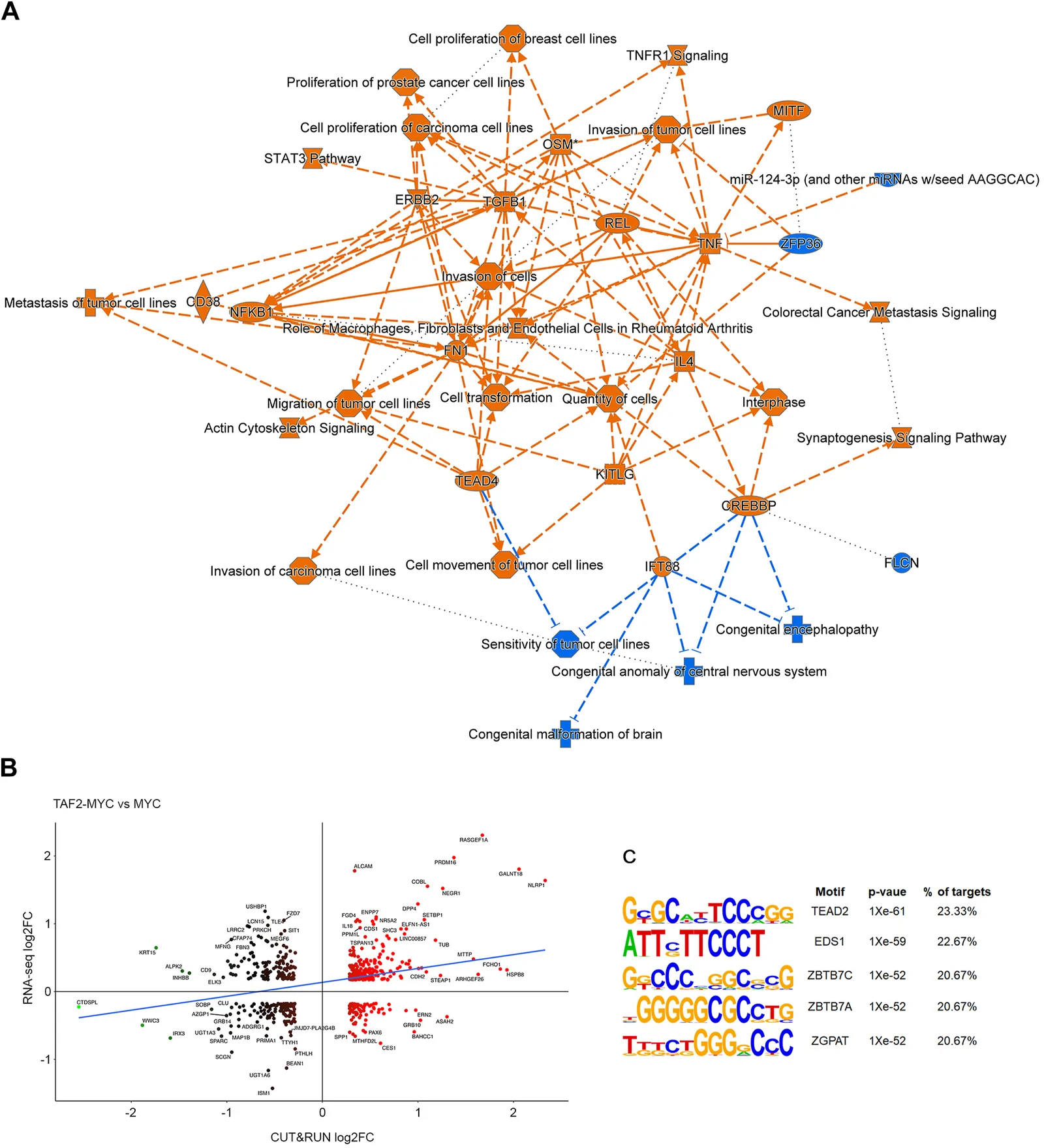
**Correlation of RNA-seq and CUT&RUN data identifies unique genes regulated by MYC in Hep-TAF2-MYC clone compared to Hep-MYC clone.***A*, graphical summary of DEGs of CUT&RUN analysis showing activation or inhibition of key regulatory molecules, pathways, functions and phenotypes in Hep-TAF2-MYC clone *versus* Hep-MYC clone. *Orange color* indicates activation z-score >2, while *blue color* indicates inhibition z-score <2. The shapes indicate the following: octagon: function; cross: disease; inverted *triangle*: kinase; ellipse: transcription regulator; *square*: cytokine; trapezoid: transporter; *circle*: molecule of other class; hour glass: canonical pathway. *B*, correlation of DEGs identified by RNA-seq and CUT&RUN analyses. Log2FC: log2 fold change. Pearson correlation: 0.52; *p*-value: 1.33e-32. *C*. Top 5 binding motifs in 18 differentially expressed gene promoters revealed by *de novo* motif analysis.

We performed correlation of log2 fold changes (FC) of RNA-seq and CUT&RUN data which unraveled four types of correlation: (*i*) positive-positive (upper right quadrant) with positive FC in both RNA-seq and CUT&RUN indicating that for these genes, MYC binds to the genes and induces their expression, (*ii*) positive-negative (lower right quadrant) with negative FC in RNA-seq and positive FC in CUT&RUN indicating that for these genes, MYC binds to the genes and inhibits their transcription, (*iii*) negative-negative (lower left quadrant), negative FC in both RNA-seq and CUT&RUN indicating that for these genes, MYC binding is decreased with corresponding decrease in transcription, and (*iv*) positive-negative (upper left quadrant): with positive FC in RNA-seq and negative FC in CUT&RUN indicating that for these genes MYC binding is decreased with an increase in their transcription which might be applicable to those genes for which MYC functions as transcription repressor (Fig. 3*B*). For further analysis, we focused on genes with positive-positive changes and refined our analysis utilizing more stringent parameters (1): inclusion of those genes to which MYC binds only to the promoters, and (2) Use of cut-off values of log2FC > 0.59 and adjusted *p*-value <0.05 for both RNA-seq and CUT&RUN datasets. This stringent analysis resulted in the identification of 18 genes that were significantly increased in Hep-TAF2-MYC clone compared to Hep-MYC clone, suggesting that in the presence of TAF2, MYC binds to these gene promoters and positively regulates their expression (Table 1). Inspection of CUT&RUN profiles at the 18 genes showed mostly higher signals of MYC binding in the Hep-TAF2-MYC clone, suggesting that TAF2 overexpression leads to higher binding of MYC at these promoters (Fig. S2). De novo motif analysis of these 18 promoters revealed a consensus DNA motif for TEA domain transcription factor 2 (TEAD2) covering the highest percentage of targets and the most significant *p*-value (Fig. 3*C*). Additional motifs for zinc finger and BTB domain containing 7C (ZBTB7C), zinc finger and BTB domain containing 7A (ZBTB7A), and zinc finger CCCH-type and G-patch domain containing (ZGPAT) were also detected by motif analysis (Fig. 3*C*).

**Table 1:** List of genes from correlation of DEGs of CUT&RUN and RNA-seq data from Hep-TAF2-MYC *versus* Hep-MYC cells with log2FC > 0.59, FDR < 0.05 and p-adj < 0.05

Gene	Description	log2FC CUT&RUN	log2FC RNA-seq
RASGEF1A	RasGEF domain family member 1A	1.6738	2.3056
PRDM16	PR/SET domain 16	3.7977	1.9754
GALNT18	polypeptide N-acetylgalactosaminyltransferase 18	2.0578	1.8053
NLRP1	NLR family pyrin domain containing 1	4.1894	1.6378
COBL	cordon-bleu WH2 repeat protein	1.4631	1.5525
NEGR1	neuronal growth regulator 1	1.6249	1.5211
DPP4	dipeptidyl peptidase 4	1.1186	1.2916
MICAL2	microtubule associated monooxygenase, calponin and LIM domain containing 2	1.0966	1.0343
KIF17	kinesin family member 17	0.6402	1.0088
SHC3	SHC adaptor protein 3	0.8227	0.9242
ELFN1-AS1	ELFN1 antisense RNA 1	0.8568	0.9241
LINC00857	long intergenic non-protein coding RNA 857	0.8598	0.8503
NIPAL1	NIPA like domain containing 1	0.6769	0.8208
VNN1	vanin 1	0.6958	0.7837
TRPV3	transient receptor potential cation channel subfamily V member 3	0.7666	0.7655
TUB	TUB bipartite transcription factor	1.1840	0.7584
BTN3A3	butyrophilin subfamily 3 member A3	0.6290	0.6966
SLC37A2	solute carrier family 37 member 2	0.6344	0.5973

Among the 18 genes, RASGEF1A was the most upregulated gene in Hep-TAF2-MYC clone compared to Hep-MYC clone (Table 1). Additionally, there was minimal MYC binding to RASGEF1A promoter in Hep-MYC and Hep-GFP clones, while the binding was significantly augmented in Hep-TAF2-MYC clone (Fig. 4*A*). Because of its role in regulating Ras signaling, we focused on our studies on RASGEF1 and Ras and downstream signaling. RASGEF1A mRNA was significantly increased in Hep-TAF2-MYC clone *versus* Hep-MYC clone (Fig. 4*B*). Rasgef1a mRNA was also significantly increased in mouse tumors generated by hydrodynamic injection of TAF2 and MYC compared to the tumors generated by MYC injection alone (Fig. 4*C*) (13). We could not find a suitable antibody for RASGEF1A and as a marker of its function we performed Ras activity assay. We observed marked increase in activated Ras in Hep-TAF2-MYC clone compared to Hep-GFP, Hep-TAF2 and Hep-MYC clones (Figs. 4, *D* and *E*, S3). Compared to Hep-GFP and Hep-TAF2 clones, an increase in activated MEK and ERK was observed in Hep-MYC clone which was further augmented in Hep-TAF2-MYC clone (Figs. 4, *F* and *G*, S3). Similar levels of AKT activation were observed in both Hep-MYC and Hep-TAF2-MYC clones compared to Hep-GFP and Hep-TAF2 clones (Figs. 4, *F* and *G*, S3). There is conflicting report about the substrate of RASGEF1A. In cholangiocarcinoma, it has been shown to activate Ras (17). However, *in vitro* binding assays documented that RASGEF1A binds specifically to RAP2, a member of the Rap subfamily of Ras-like G-proteins (18). Knocking down RASGEF1A by siRNA decreased the levels of active Ras in Hep-TAF2-MYC cells suggesting that similar to cholangiocarcinoma cells, RASGEF1A might promote Ras activation as well (Figs. 4, *H*–*J* and S3). As a corollary, downregulation of RASGEF1A resulted in a decrease in MEK/ERK activation in Hep-TAF2-MYC clone (Figs. 4, *K–L* and S3). Compared to control siRNA, RASGEF1A siRNA significantly inhibited viability of Hep-TAF2-MYC clone (Fig. 4*M*). De novo motif analysis identified a consensus TEAD2 motif in RASGEF1A gene promoter (Fig. 3*C*). Knocking down TEAD2 by siRNA significantly decreased the levels of RASGEF1A mRNA in Hep-TAF2-MYC cells suggesting a potential contribution of TEAD2 in TAF2+MYC-mediated regulation of RASGEF1A transcription (Fig. 4*N*).

**Figure 4:**
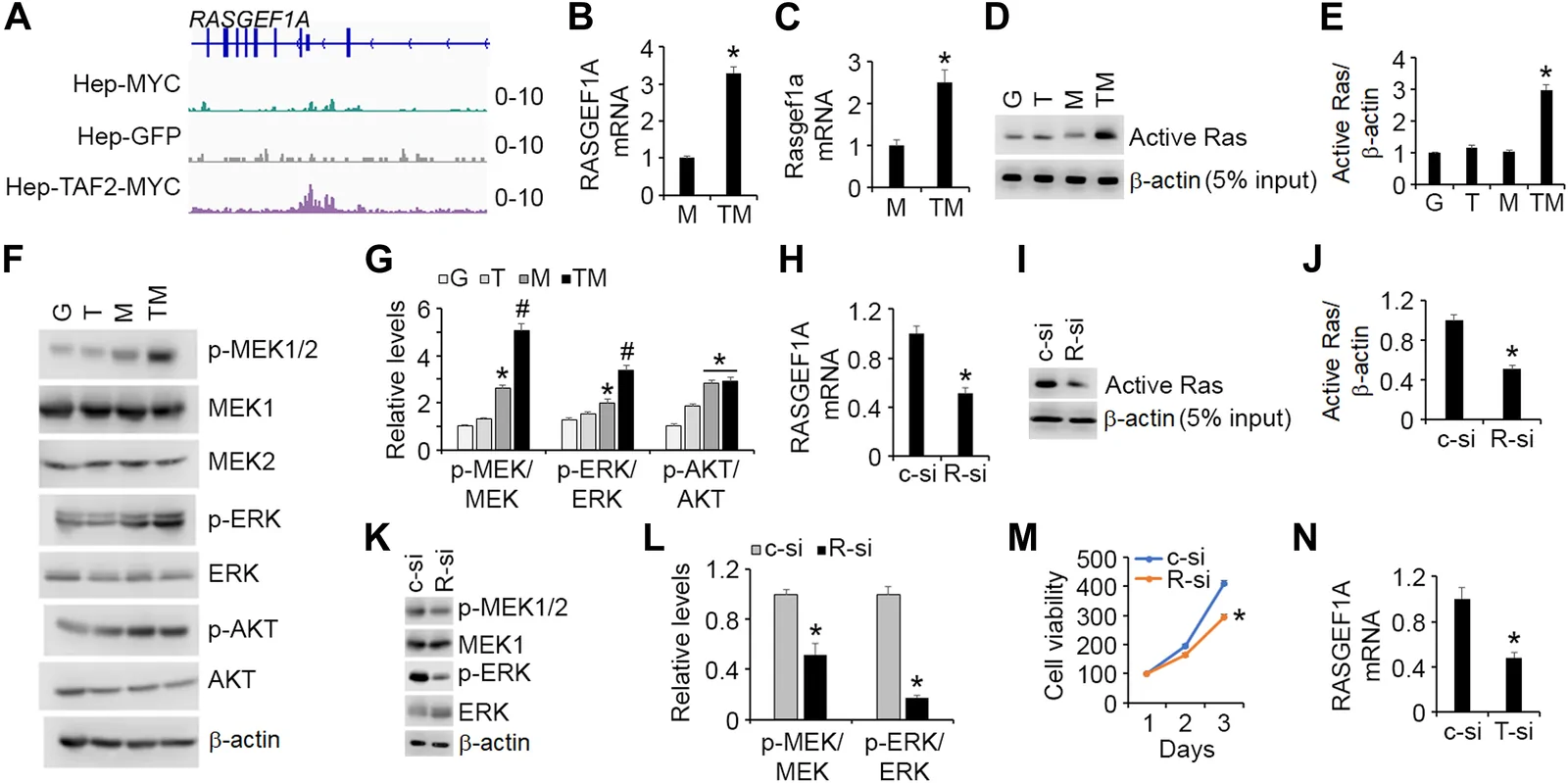
**TAF2 and MYC overexpression induces RASGEF1A and activates Ras downstream signaling.** G, Hep-GFP; M, Hep-MYC; T, Hep-TAF2; TM, Hep-TAF2-MYC. *A*, representative histogram of MYC binding to the promoter region of RASGEF1A gene in the indicated cells. CUT&RUN was performed using anti-HA antibody. *B*, RASGEF1A mRNA expression in the indicated cells. *C*, Rasgef1a mRNA expression in mouse tumors generated by hydrodynamic injection of MYC (M) or TAF2 and MYC (TM). N = 5 per group. For *B* and *C*, data represent mean ± SEM. ∗: *p* < 0.01. *D*, Representative Western blot of Ras activity assay in the indicated cells. *E*, quantification of Western blot presented in (*D*). Data represent mean ± SEM. ∗: *p* < 0.01 *versus* all other groups. *F*, representative Western blots of the indicated proteins in the indicated cells. β-actin was used as a loading control. *G*, Quantification of Western blot presented in *F*. Data represent mean ± SEM. ∗: *p* < 0.05 *versus* G & T; #: *p* < 0.05 *versus* G, T & M. H. RASGEF1A mRNA levels in Hep-TAF2-MYC clone transfected with control siRNA (c-si) or RASGEF1A siRNA (R-si) for 48 h. Data represent the mean ± SEM. ∗: *p* < 0.01. I. Cells were treated as in *H*, and Ras activity assay was performed. *J*, quantification of the Western blot presented in *I*. Data represent mean ± SEM. ∗: *p* < 0.01. *K*. Cells were treated as in *H*, and Western blots were performed for the proteins as indicated. *L*, Quantification of Western blot presented in *K*. Data represent mean ± SEM. ∗: *p* < 0.01. *M*, Cell viability assay in Hep-TAF2-MYC clone transfected with control siRNA (c-si) or RASGEF1A siRNA (R-si) at the indicated time points. Data represent mean ± SEM. ∗: *p* < 0.01. *N*, RASGEF1A mRNA levels in Hep-TAF2-MYC clone transfected with control siRNA (c-si) or TEAD2 siRNA (T-si) for 48 h. Data represent mean ± SEM. ∗: *p* < 0.01.

We checked the sensitivity of the clones to MEK inhibitor trametinib and AKT inhibitor MK2206. The IC50 for Trametinib ranged between 0.5408 to 0.6263 nM for Hep-GFP, Hep-TAF2 and Hep-MYC clones while it was 0.0739 nM for Hep-TAF2-MYC clone (Fig. 5). More importantly, at higher doses, Trametinib could completely kill Hep-TAF2-MYC clone but not the other three clones (Fig. 5). All four lines showed similar sensitivity to AKT inhibitor MK2206 (Fig. S4). These findings indicate that the heightened activation of MEK/ERK pathway in Hep-TAF2-MYC clone conferred a unique sensitivity to MEK inhibitor Trametinib.

**Figure 5:**
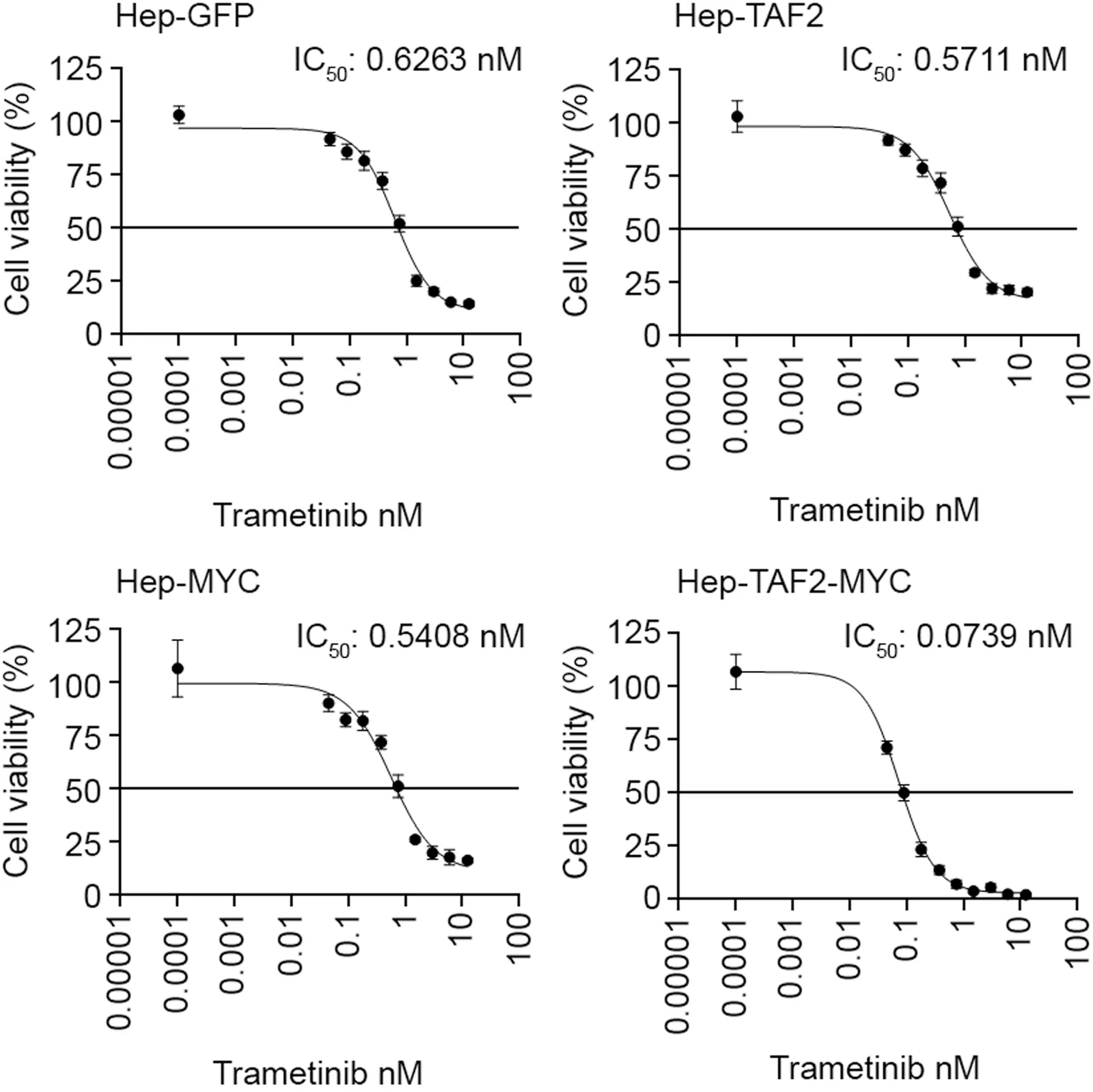
**TAF2 and MYC overexpression in HepG2 cells confers unique sensitivity to MEK inhibitors.** Cell viability assay in the indicated cells after treating with trametinib at 0.0488, 0.098, 0.195, 0.39, 0.78, 1.5625, 3.125, 6.25, and 12.5 nM doses for 3 days. Data represent mean ± SEM. Compared to vehicle-treated cells, all treated datapoints showed a *p*-value < 0.0001.

## Discussion

In this manuscript, we endeavored to untangle the molecular mechanism by which TAF2 modulates MYC-mediated gene regulation to promote MYC-induced hepatocarcinogenesis. While overexpression of either TAF2 or MYC promoted proliferation, invasion, and migration of HepG2 cells, these effects were further augmented upon co-overexpression. Notably, hydrodynamic injection in naïve mouse liver revealed that only MYC is sufficient to induce tumor formation (13), suggesting that TAF2 lacks independent transforming properties. Instead, TAF2 augments cancer hallmarks only within a previously transformed cellular context. Once MYC transforms a cell, TAF2 cooperates to shift the MYC-mediated transcriptional landscape toward additional oncogenic targets, thereby significantly amplifying the malignant phenotype.

CUT&RUN analysis revealed that in Hep-MYC cells, MYC binding to the promoter regions (∼2 kb upstream of the transcription start site; Promoter.Up), introns, and distal intergenic regions was 26.1%, 29.7% and 26.9%, respectively. In Hep-TAF2-MYC cells, MYC binding at the same sites shifted to 51.5%, 18% and 13.5%, respectively. This observation suggests that TAF2 overexpression steers MYC towards promoter regions where TAF2 resides as part of the TFIID complex. MYC has been shown to directly interact with TBP, with mutants that abrogate MYC-TBP interaction exhibiting compromised MYC activity (19). We now show that MYC also interacts with TAF2 to modulate and augment MYC-induced oncogenic phenotypes (Fig. 6).

**Figure 6:**
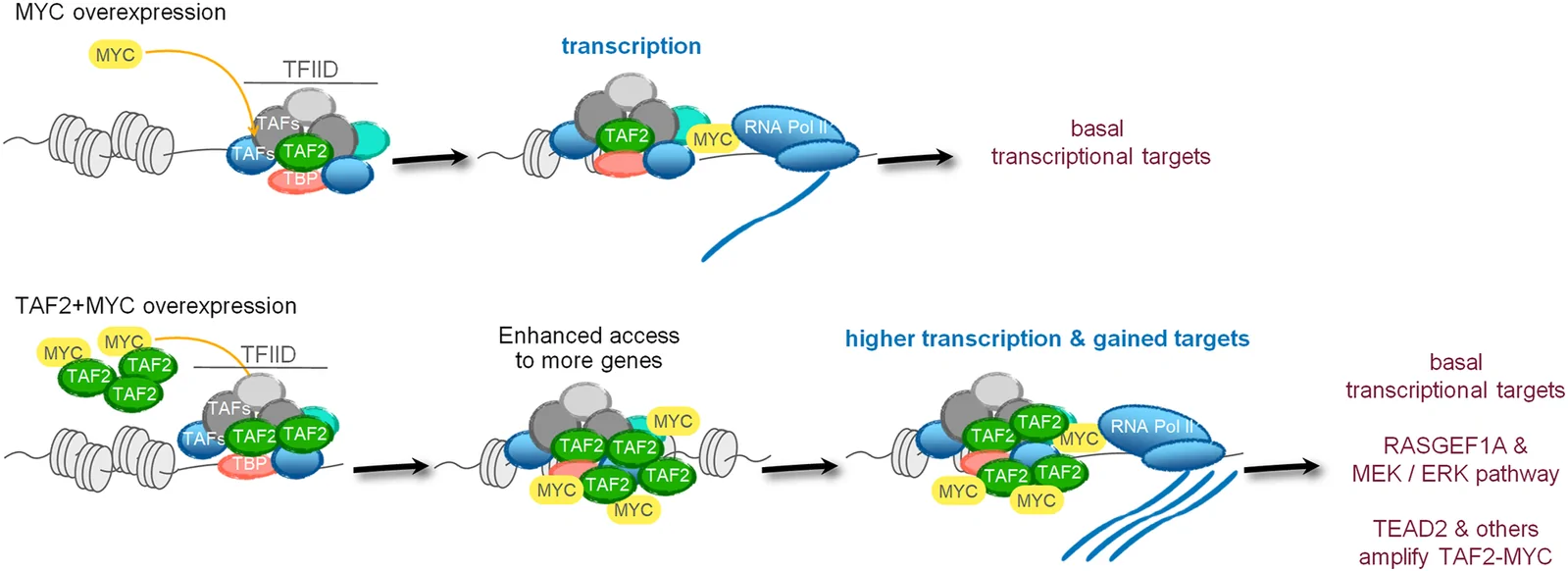
**Cartoon showing potential mechanism of cooperation between TAF2 and MYC.** Overexpressed TAF2 facilitates overexpressed MYC binding to promoters of specific genes, such as RASGEF1A, with TEAD family proteins amplifying this effect. This leads to activation of MEK/ERK pathway that confers sensitivity to MEK inhibitors to TEF2+MYC-overexpressing tumors.

Using stringent parameters for analysis, we identified 18 genes whose promoters are differentially bound by MYC in the Hep-TAF2-MYC clone, leading to induced expression. These 18 genes have established oncogenic properties. RasGEF domain family member 1A (RASGEF1A) and SHC adaptor protein 3 (SHC3) are known to activate the oncogenic Ras signaling pathway (17, 18, 20). PR/SET domain 16 (PRDM16) is a transcription factor acting as an oncogene in HCC (21). Polypeptide N-acetylgalactosaminyltransferase 18 (GALNT18), which catalyzes protein O-glycosylation has been shown to augment HCC in mouse models (22). HCC results from chronic inflammation and sustained activation of inflammasomes, which contain NLR family pyrin domain containing 1 (NLRP1), contributing to HCC development and progression (23). Dipeptidyl peptidase 4 (DPP4/CD26), a serine protease known to cleave chemokines, is upregulated in HCC and DPP4 inhibitors were shown to inhibit HCC by enhancing natural killer (NK) and T cell-mediated killing of HCC cells (24). This gene set further includes long intergenic non-protein coding RNA 857 (LINC00857), an oncogenic lncRNA in multiple cancers, including HCC, where it promotes epithelial-mesenchymal transition (EMT) (25). Similar EMT-promoting properties have also been shown for microtubule-associated monooxygenase, calponin and LIM domain containing 2 (MICAL2) (26). We also identified cordon-bleu WH2 repeat protein (COBL), neuronal growth regulator 1 (NEGR1) and kinesin family member 17 (KIF17), the function of which remains to be shown in HCC, thereby providing new avenues for study.

De novo motif analysis of the 18 gene promoters discovered a consensus TEAD2 motif (Fig. 3*C*). The TEAD family of proteins share a consensus motif, and interestingly, TEAD4, which promotes EMT and metastasis in liver cancer (27), was identified to be activated in Hep-TAF2-MYC clone (Fig. 3*A*). In liver, there is a unique relationship between TEAD and MYC, where constitutive binding of TEAD has been observed at a large fraction of MYC target genes (28). In low MYC conditions, TEAD heterodimer partner YAP (Yes1-associated transcriptional regulator) is bound to its canonical target. When MYC is overexpressed, TEAD facilitates recruitment of YAP to MYC target genes, driving full transcriptional activation and consequently cell cycle entry, organ growth and tumorigenesis (28). The concomitant overexpression of MYC and TAF2 might accentuate this process further, by stabilizing the recruitment of the TFIID complex to these sites. Indeed, knocking down TEAD2 significantly decreased RASGEF1A mRNA levels in Hep-TAF2-MYC cells (Fig. 4*N*). Further studies involving a detailed promoter assembly of transcription factors and structural biology will be needed to validate these observations and further define the biochemical basis of this cooperation. Additional future direction includes performing domain mapping studies to identify interface of TAF2/MYC interaction, crystalize that region and develop small molecule inhibitors that disrupt TAF2/MYC interaction. These inhibitors might potentially exert anti-cancer effect in TAF2- and MYC-overexpressing tumors.

## Experimental procedures

### Generation of lentivirus, establishment of stable clones, and culture conditions

To generate the pLenti/CMV/Hygro/MYC-HA construct the human MYC ORF in c-myc-PT3EF1a (Addgene plasmid 92,046) was sequentially cloned into the pENTR/D-TOPO and pLenti/CMV/Hygro/DEST (w117-1) vectors using Invitrogen pENTR Directional TOPO and Gateway LR Clonase kits. A G4S linker followed by an HA tag was added to the MYC C-terminus during cloning. To generate the pLenti/CMV/Puro/TAF2 (V5/His6) construct the human TAF2 ORF (from Genomics-online ABIN4944851) was initially cloned into the pTRACER/EF1alpha vector, replacing the C-terminal FLAG tag with C-terminal V5 and His6 tags. The TAF2 (V5/His6) ORF was then sequentially cloned into the pENTR/D-TOPO and pLenti/CMV/Puro/DEST (w118-1) vectors using Invitrogen pENTR Directional TOPO and Gateway LR Clonase kits. HepG2 cells were transduced with Lenti/CMV/Puro/eGFP or Lenti/CMV/Puro/TAF2 (V5/His6) and pooled clones were selected using 1 μg/ml of puromycin. HepG2 cells were transduced with Lenti/CMV/Hygro/eGFP or Lenti/CMV/Hygro/MYC-HA, and pooled clones were selected using 200 μg/ml of hygromycin. Both puromycin- and hygromycin-resistant GFP-overexpressing cells proliferated at a similar manner, and as such, puromycin-resistant GFP-overexpressing clone (Hep-GFP) was used as a control. TAF2- and MYC- overexpressing HepG2 clones were termed Hep-TAF2 and Hep-MYC, respectively. Hep-TAF2 clone was transduced with Lenti/CMV/Hygro/MYC-HA, and pooled clones were selected using 400 μg/ml of hygromycin to generate TAF2+MYC-overexpressing clone (Hep-TAF2-MYC). All cells were *mycoplasma-free* as detected by the *Mycoplasma* Detection Kit (ThermoFisher). Hep-TAF2-MYC cells were transduced with either control siRNA, RASGEF1A siRNA or TEAD2 siRNA (Santa Cruz Biotechnology, catalog# sc-37007, sc-90732 and sc-45232, respectively) using lipofectamine 2000 according to the manufacturer’s protocol. RASGEF1A siRNA is a pool of 3 different siRNA duplexes (5′: CCAGACAGAUCCAUGAGUU: 3′; 5′: CUAGUCUUGAAACCUAUCA: 3′; 5′: CUAGUACCUACUUGAAACA: 3′). TEAD2 siRNA is a pool of 3 different siRNA duplexes (5′: GAUGUAUGGUCGGAAUGAA: 3′; 5′: CAGAUGCAGUUGAUUCUUA: 3′; 5′: GUGUUCUGACAGAGGAUAA: 3′). Cells were treated with different concentrations of Trametinib and MK2206 (both obtained from Selleckchem.com) and cell viability was determined by MTT assay 72 h later. HuH-7 cells were developed in Japan and were obtained from Japanese Collection of Research Bioresources Cell Bank.

### Total RNA extraction, cDNA preparation, quantitative RT-PCR (Q-RT-PCR), and RNA sequencing (RNA-seq)

Total RNA was extracted using the QIAGEN miRNeasy Mini Kit (QIAGEN). cDNA preparation was done using ABI cDNA synthesis kit (Applied Biosystems). Q-RT-PCR was performed using an ABI ViiA7 fast real-time PCR system and Taqman gene expression assays according to the manufacturer’s protocol (Applied Biosystems, Foster City, CA). Total RNA, extracted from the Hep-MYC and Hep-TAF2-MYC cells, was employed for RNA-Seq (13). RNA-Seq library was prepared using Illumina TruSeq RNA sample preparation kit and subjected to two rounds of sequencing on Illumina HiSeq3000 platform. RNA-Seq libraries were pooled together to aim for about 25-40M read passed filtered reads per sample. All sequencing reads were processed using the Nextflow rnaseq v.23.04.3 pipeline using the hg38 or mm10 genome assemblies for human and mouse genomes, respectively, and the star_rsem gene quantification method. The differential expression analysis was performed using edgeR v3.18.1. Genes having counts per million less than two in all samples were excluded. Differentially expressed genes were defined using False Discovery Rate (FDR) < 0.01 cutoffs. All bioinformatics analyses were conducted in R/Bioconductor computing environment v3.4.0. Processed gene expression data is available on GEO (GSE327282, reviewer's access token kpalyeaifzgvzat).

### Cleavage under targets & release using nuclease (CUT&RUN)

CUT&RUN was performed in Hep-GFP, Hep-MYC, and Hep-TAF2-MYC clones with anti-HA antibody, using previously described protocol (29). Briefly, nuclei were isolated by incubating cells on ice for 5 min in Buffer A with protease inhibitor and 0.1% Triton X-100. After centrifugation at 1750*g* for 2 min at 4 °C, nuclei were resuspended in Wash buffer. Bio-Mag Plus Concanavalin-A (Con A) coated beads (Bangs Laboratories BP531) activated in Binding buffer were then added to the nuclei and rotated for 10 min at room temperature. Primary antibody (0.5 μg) was added to the bead-nuclei mixture and incubated for 2 h at room temperature. Beads were washed twice with Digitonin buffer and incubated with pAG-MNase for 10 min at room temperature. Beads were then washed once with Digitonin buffer, incubated with 2 mM CaCl_2_ for 15 min at room temperature, and quenched by adding Stop buffer. DNA was released from the beads by incubating them for 15 min at 37 °C and purified by CUTANA DNA purification kit (EpiCypher SKU:14-0050). Libraries were constructed using xGen ssDNA & Low-Input DNA Prep by following the manufacturer’s instructions (IDT 10009817) and sequenced at the St Jude Hartwell Center. CUT&RUN Wash buffer: 20 mM HEPES (pH 7.5), 150 mM NaCl, 0.5 mM spermidine, protease inhibitor cocktail (Sigma-Aldrich 11873580001). CUT&RUN Binding buffer: 20 mM HEPES-KOH (pH 7.9), 10 mM KCl, 1 mM CaCl2, 1 mM MnCl2. CUT&RUN Digitonin buffer: CUT&RUN Wash buffer with 0.01% digitonin. CUT&RUN Antibody buffer: CUT&RUN Digitonin buffer with 2 mM EDTA. CUT&RUN 2X Stop buffer: 340 mM NaCl, 20 mM EDTA, 4 mM EGTA. CUT&RUN Stop buffer: Into 1 ml of 2X Stop buffer stock, 5 μl of 10 mg/ml and 3.3 μl of 15 mg/ml RNase A was added.

### Analysis of CUT&RUN

50 bp single-end reads were obtained and aligned to human genome hg38 by BWA (version 0.7.170.7.12, default parameter). Uniquely mapped reads were kept by samtools (parameter “-q 1 -F 1804,” version 1.14). Fragments < 2000 bp were kept for peak calling, and bigwig files were generated for visualization. Macs2 was used for peak calling to identify peaks. Strong peaks were called with parameter ‘FDR < 0.05′ and weak peaks were called with parameter ‘FDR < 0.5′. Peaks were considered reproducible if they were supported by one strong peak and at least one weak peak in other replicates. For downstream analyses, heatmaps were generated by deepTools and gene ontology was performed with Enrichr and GSEA, in addition to custom R scripts. For differential peak analysis, peaks from two replicates were merged and counted for number of overlapping extended reads for each sample (bedtools v2.24.0), and we detected the differential peaks by the empirical Bayes method (eBayes function from the limma R package). Peaks were annotated based on Gencode following this priority: “Promoter.Up”: if they fall within TSS - 2 kb, “Promoter.Down”: if they fall within TSS - 2 kb, “Exonic” or “intronic”: if they fall within an exon or intron of any isoform, “transcriptional end site peaks”: if they fall within site ± 2 kb, “distal5” or “distal3” if they are with 50 kb upstream of TSS or 50 kb downstream of transcriptional end site, respectively, and they are classified as “intergenic” if they do not fit in any of the previous categories. Promoter sequences of the 18 genes directly altered by MYC-HA were used to identify *de novo* motifs overrepresented relative to background sequences. Motif enrichment analysis was performed using the findMotifsGenome.pl script and the Homer motif discovery algorithm (30).

### Correlation analysis

To identify genes with concordant differential expression across two comparisons, we analyzed the agreement of log2 fold changes from each condition. These values were plotted on an XY scatter plot, and genes exhibiting positive or negative fold changes in both conditions were selected. The log2 fold changes were then converted to ranks, and genes were prioritized based on their average rank, with ranks reversed for genes displaying negative fold changes. This approach ensures that genes with the most extreme positive or negative fold changes in both conditions are given the highest priority.

### Ras activity assay

Ras activity assay was performed using a kit from Cell Signaling Technology (Catalog#8821) according to the manufacturer’s instruction. Briefly, 500 μg of cell lysate was mixed with 80 μg GST-Raf1-RBD protein and 100 μl 50% slurry of glutathione-agarose beads in a spin cup at 4 °C for 1 h. Following three washes with the lysis buffer, 1X SDS sample buffer was used for elution and the eluates were subjected to Western blot using anti-Ras antibody (Cell Signaling Technology; mouse monoclonal; 1:20 dilution). Five per cent of each input was used for Western blot using anti-β-actin antibody (Cell Signaling Technology; mouse monoclonal; 1:1000 dilution).

### MTT assay

Cells (2 × 10^3^) were plated in each well of a 96 well plate in 100 μl media. At the desired time point, media was replaced with 3-(4,5-Dimethylthiazol-2-yl)-2,5-Diphenyltetrazolium Bromide (MTT) containing media. After 5 h, MTT containing media was carefully removed and 100 μl of DMSO was added. The plates were placed on a shaker for 5 to 10 min at full speed to dissolve the crystals. Absorbance was measured at 600 nm using Promega Glomax Discover plate reader.

### Colony formation assay

Two hundred fifty (250) cells were plated in a 6 cm dish in 5 ml media. After 3 weeks, cells were fixed with 3.7% formaldehyde and stained with 0.5% crystal violet. The number of colonies (>50 cells) were counted using ImageJ software (https://imagej.net/ij/). Colony-forming efficiency was calculated as (Number of colonies counted/Number of cells plated) x 100.

### Migration assay

Cells (3 × 10^6^/ml) were plated in a 12-well dish and allowed to reach 80% confluency. Confluent cells were scratched with a 200 μl micropipette tip to create a cell-free area. The scratched monolayer was imaged at different time points after wounding. A mark was made on the plates to make sure the same area was being imaged every day. Images were obtained at 50x and 100x using a phase contrast microscope. The wound distance area was measured using the Image J software Bandpass Filter function in μm. Wound healing rate was calculated as % of initial wound distance (wound distance at time series/initial time × 100%).

### Invasion assay

An invasion assay was performed using Corning Biocoat Matrigel Invasion chambers in a 24-well plate. The chambers were allowed to rehydrate for 2 hours at 37 °C. The lower chamber was filled with complete media (containing 10% FBS), and 5 × 10^5^ cells were seeded in the upper chamber in serum-free medium. The cells for the invasion assay were incubated at 37 °C in a 5% CO_2_ humidified incubator. After 22 h, cells in the upper chambers were removed by wiping with a cotton swab, and invasion was determined by fixing with 100% methanol, staining with 0.5% crystal violet and manually counting the number of cells that have migrated to the lower chamber under a Zeiss microscope. Images were obtained at 100X magnification.

### Western blot, co-immunoprecipitation, and immunofluorescence (IF) assays

Cell lysates were prepared, and Western blotting and co-immunoprecipitation were performed as described (31, 32). Images of the full blot with the molecular sizes of the proteins are shown in Fig. S3. For Western blot the following primary antibodies from Cell Signaling Technology were used: anti-V5 (rabbit, 1:1000), anti-HA (rabbit, 1:1000), anti-β-actin (mouse monoclonal, 1:1000), anti-MEK1 (mouse monoclonal, 1:1000), anti-MEK2 (rabbit, 1:1000), anti-phospho-MEK1/2 (rabbit, 1:1000), anti-ERK (rabbit, 1:1000), anti-phospho-ERK (rabbit, 1:1000), anti-AKT (rabbit, 1:1000), anti-phospho-AKT (rabbit, 1:1000), anti-c-MYC (rabbit, 1:1000), anti-TBP (rabbit, 1:1000), anti-Vinculin (rabbit, 1:1000). Anti-GAPDH (mouse monoclonal, 1:2000), anti-c-Myc (mouse monoclonal, 1:500) and anti-TAF8 (mouse monoclonal, 1:500) antibodies were obtained from Santa Cruz Biotechnology. Anti-TAF2 antibody (mouse monoclonal, 1:500) was generated in-house (13). For co-IP, the primary antibodies were used at 1:100 dilution. Western blots were quantified using ImageJ software (https://imagej.net/ij/). IF was performed as described (31) using anti-V5 and anti-HA antibodies.

### Statistical analysis

Data were presented as the mean ± Standard Error of Mean (S.E.M) and analyzed for statistical significance using Tukey’s multiple comparisons test of One and Two-way ANOVA by GraphPad Prism, version 9 software (GraphPad Software, Inc).

## Data availability

All data are contained within the manuscript. The GEO Series accession number of the RNA-seq data is GSE327282 (reviewer's access token kpalyeaifzgvzat). The GEO Series accession number of the CUT&RUN dataset is GSE329914 (reviewer token: not needed).

## Supporting information

This article contains supporting information.

## Conflict of interest

The authors declare that they have no conflicts of interest with the contents of this article.

## Figures and Tables

**Figure S1:**
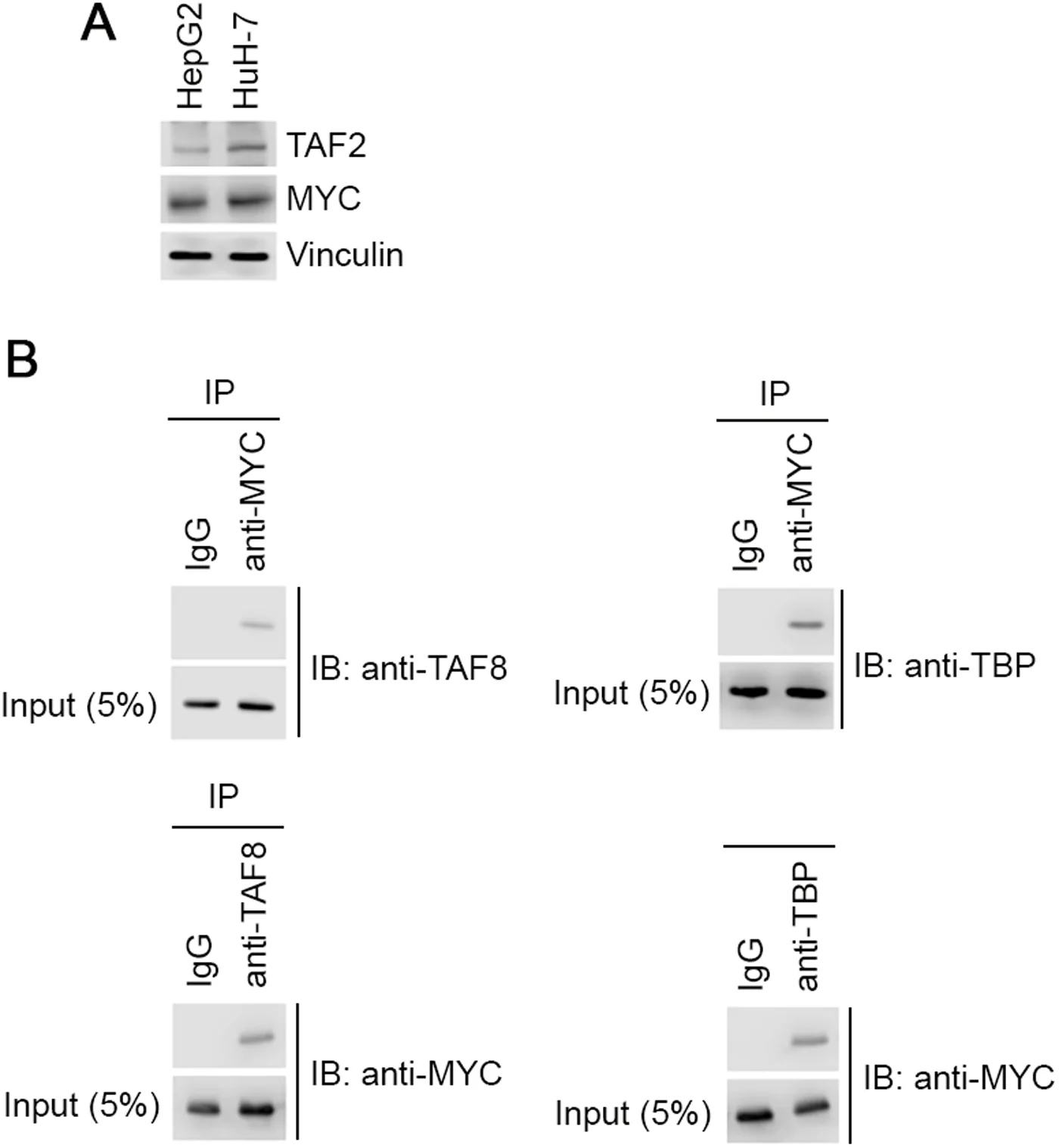


**Figure S2:**
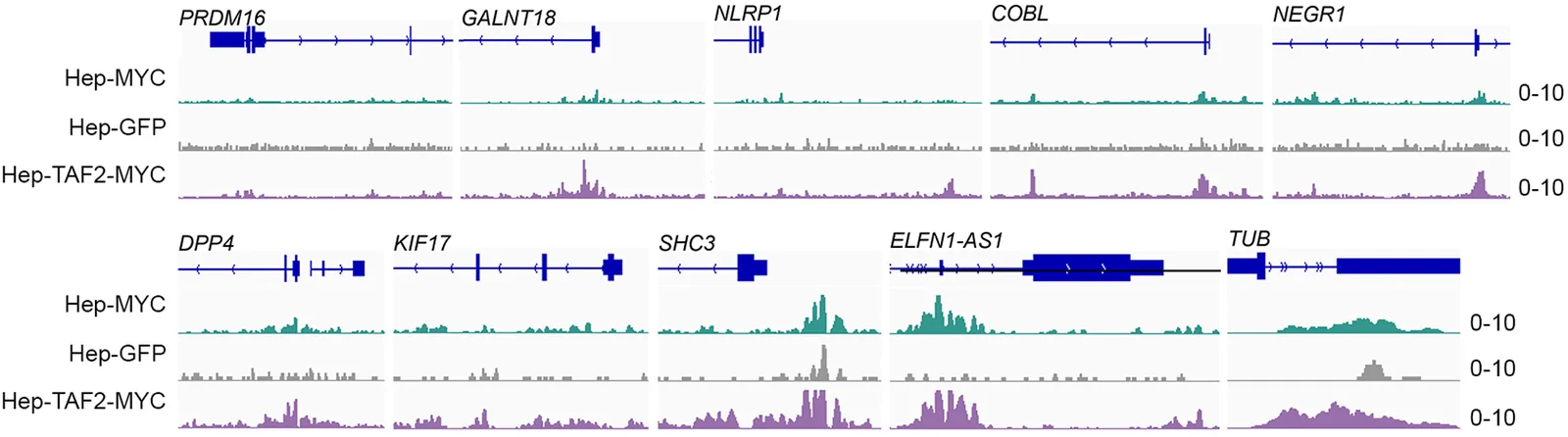


**Figure S3:**
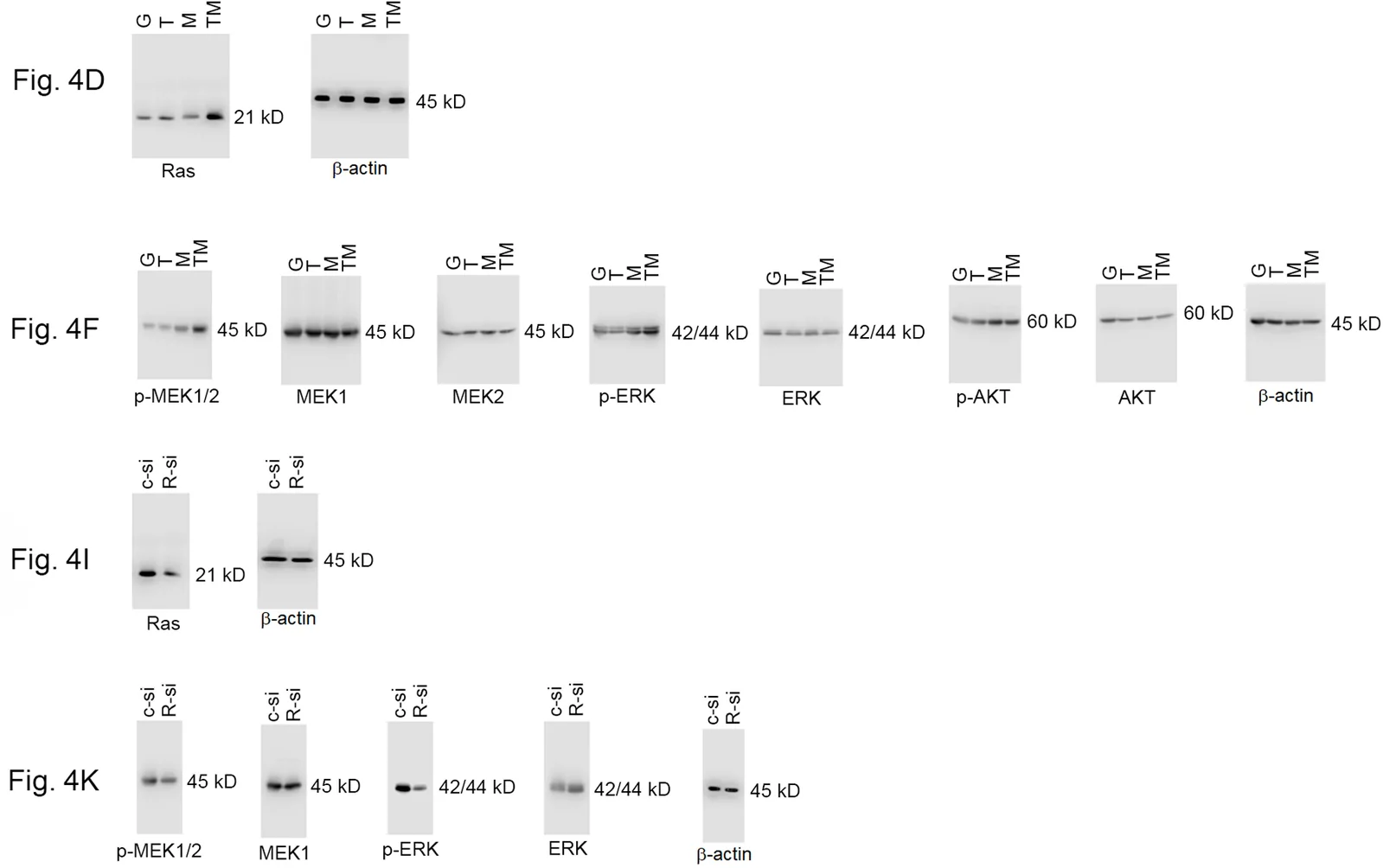


**Figure S4:**