# Supplementary material for: Molecular mechanism of augmentation of oncogenic activity of MYC by TAF2 in HCC
Source: J Biol Chem. 2026 Sep 18;302(11):113581. doi: 10.1016/j.jbc.2026.113581 (PMC13682000; doi:10.1016/j.jbc.2026.113581)
Supplement: Supplementary file 1 [file mmc1.docx]

**Legends to supplementary tables and figures**

Table S1. List of differentially expressed genes (DEGs) identified by RNA-seq in Hep-TAF2-MYC clone vs Hep-MYC clone using a log2 fold-change (log2FC) of 0.5 and false discovery rate (FDR) of 0.01.

Table S2. List of genes and binding sites identified by CUT&RUN showing differential binding of MYC in Hep-TAF2-MYC clone vs Hep-MYC clone using a log2FC of 1 and an adjusted p-value of 0.05. The genes starting with RF represent SNO RNAs.

Figure S1. A. Representative Western blot showing the levels of indicated proteins in the indicated cell lines. Vinculin was used as loading control. B. Lysates from HuH-7 cells were immunoprecipitated (IP) with anti-MYC antibody and immunoblotted (IB) with anti-TAF8 and anti-TBP antibodies and vice versa. Representative images shown.

Figure S2. Representative histograms of MYC binding to the promoter regions of the indicated genes in the indicated cells. CUT&RUN was performed using anti-HA antibody.

Figure S3. Images of full Western blots presented in Fig. 4.

Figure S4. Cell viability assay in the indicated cells after treating with MK2206 at 0.15, 0.3, 0.6, 1.25, 2.5, 5, 10 and 20 μM doses for 3 days. Data represent mean ± SEM. Compared to vehicle-treated cells, only 10 and 20 μM MK2206-treated datapoints showed a p-value<0.0001.
